# Patterns of radioiodine uptake combined with pulmonary nodule size in predicting long-term clinical outcomes of differentiated thyroid carcinoma patients with lung metastasis alone

**DOI:** 10.22038/aojnmb.2025.87406.1628

**Published:** 2026

**Authors:** Mai H Thong, Le Q Khanh, Tran B Khoa, Nguyen Th Phuong, Le Ng Ha

**Keywords:** Differentiated thyroid carcinoma, Lung metastasis, Patterns of lung radioiodine uptake, Pulmonary nodule size, Prognosis

## Abstract

**Objective(s):**

This study aimed to evaluate the role of radioiodine uptake patterns and pulmonary nodule size in predicting progression- free survival (PFS) and overall survival (OS) in DTC patients with lung metastases only.

**Methods:**

A retrospective study was conducted on 189 DTC patients with pulmonary metastasis alone who were treated and monitored at the Department of Nuclear Medicine, Hospital 108, Vietnam from January 2004 to December 2018. They were categorized based on radioiodine uptake patterns on post-therapy whole-body scans (WBS) and CT-based pulmonary nodule sizes. Prognostic factors were identified by Kaplan-Meier survival and Cox regression.

**Results:**

The 5-year and 10-year OS rates were 96.83 % and 94.71 %. DTC-LM patients with diffuse radioiodine uptake and miliary metastases demonstrated significantly higher survival rates, with 5-year and 10-year PFS rates of 100% and 98.8%, respectively. In contrast, patients with nonavid metastases exhibited a markedly lower prognosis, with 5-year and 10-year PFS rates of 58.8% and 25.2%, respectively (p<0.001). Patients with nodules <5 mm had 5-year and 10-year PFS rates of 88.8% and 73.8%, respectively, while those with nodules 5–10 mm had rates of 79.8% and 52.7%. Patients with nodules ≥10 mm experienced the worst outcomes, with 5-year and 10-year PFS rates of 70.6% and 29.3% (p<0.001). Multivariate analysis confirmed that non-avid metastases, nodule size ≥10 mm, and age ≥55 were independent predictors of poor prognosis (HR:24.99, 5.96, and 6.58; p<0.001).

**Conclusions:**

Patterns of radioiodine uptake and pulmonary nodule sizes on CT imaging are crucial predictors of long-term clinical outcomes in DTC-LM. Diffuse iodine uptake and military pulmonary metastases are associated with favorable outcomes, while non-avid lung nodules and larger nodule sizes predict poorer survival.

## Introduction

 Differentiated thyroid carcinoma (DTC), which includes both papillary and follicular carcinomas, is the most prevalent form of endocrine malignancy. While the overall prognosis of DTC is typically favorable, it becomes significantly less favorable in cases where distant metastases are present (1, 2). 

 Among the various sites of distant metastasis, the lungs are the most commonly affected, with pulmonary involvement occurring in 2–20% of DTC patients (3, 4). For those DTC patients with lung metastases (DTC-LM), the prognosis is generally poorer, as evidenced by reduced survival rates when compared to patients without pulmonary metastasis (5, 6). The radiographic findings of DTC-LM are essential for assessing treatment response and predicting outcomes, with distinct patterns of radioiodine uptake observable on whole-body scans (WBS) and chest computed tomography (CT) (6-9). Based on patterns of lung radioiodine uptake, DTC-LM patients were divided into different subgroup as iodine-avid bilateral lung diffuse, lung focal uptakes or non-iodine avid metastases (6, 10). Pulmonary metastases from DTC have also been classified based on the appearance on CT imaging as miliary, micronodular or macronodular ones (4, 6). While ^131^I post-therapy WBS is an efficient functioning modality in detection of pulmonary iodine avid metastases, chest CT as an added tool depicts detailed visualization of pulmonary metastasis over sub-centimeter sizes providing incremental diagnostic and prognostic information for further management of DTC-LM patients (11, 12).

 Several previous studies conducted in limited patient cohorts have demonstrated that the classification of DTC-LM based on patterns observed in ^131^I post-therapy WBS were closely associated with the response to radioiodine therapy. In contrast, the size of metastatic pulmonary nodules, as seen on CT imaging, is considered an overall prognostic factor. Specifically, it has been reported that DTC patients exhibiting metastatic pulmonary macronodules (≥10 mm in diameter) detected on chest X-ray or CT imaging are less likely to achieve a complete response to radioiodine treatment and are associated with poorer clinical outcomes compared to DTC-LM subgroups presenting micronodules (<10 mm) or miliary metastases with detectable lung radioiodine uptake (6, 13, 14). Different subgroups of DTC-LM patients with various radiographic features are frequently encountered in clinical practice. However, only a few studies analyzed lung radioiodine concentration on WBS associated with conventional CT imaging and progression/overall survival in DTC patients with pulmonary metastasis alone (3, 6, 15).

 Therefore, the aim of our study is to determine radiographic patterns of radioiodine uptake in relation to pulmonary nodule size in predicting long-term clinical outcomes of DTC patients with lung metastases only.

## Methods

### Patient’s population

 A retrospective analysis was conducted on the medical records of 329 patients with DTCLM who were treated and monitored at the Department of Nuclear Medicine, Hospital 108, between January 2004 and December 2018. Among them, 17 patients did not undergo CT before and after ^131^I treatment. 29 DTC-LM patients having at least one other metastasis, and 31 DTCLM patients who did not receive any further regular follow-ups were excluded. To ensure a meaningful evaluation of long-term outcomes and an adequate therapeutic response, patients who received fewer than three consecutive courses of radioiodine therapy were excluded from our analysis, as mentioned in several previous studies (16, 17). 

**Figure 1:**
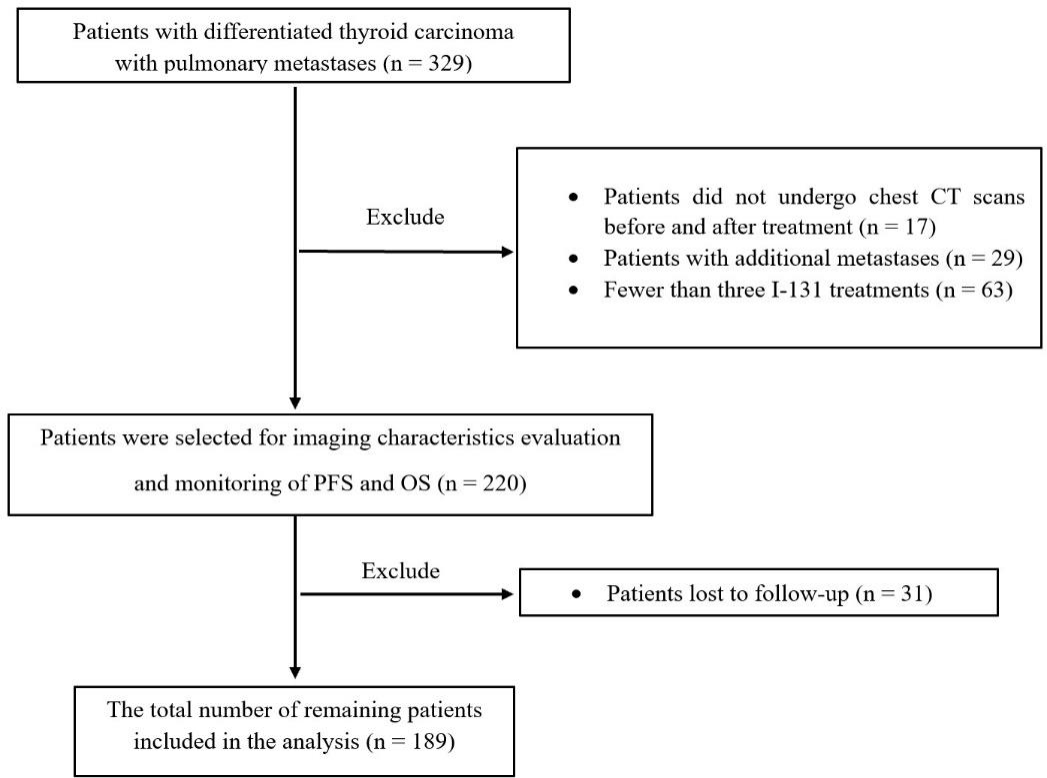
Flowchart of DTC patients with lung metastasis only were selected in present study

 This resulted in the exclusion of 63 patients. Consequently, a total of 189 DTC patients with lung metastasis only who fully met the study criteria were included in the study analysis (Figure 1). The present study was approved by the ethics committee of Hospital 108.

 Clinical data including gender, age at diagnosis of primary DTC, age at diagnosis of lung metastasis, age at initial radioiodine therapy (RIT), time of diagnosis of lung metastasis, pathological type, size of lung nodules, site of distant metastasis, and the serum Tg and anti-Tg levels after TSH stimulation prior to the initial RIT, were collected from medical records.

Follow-up data included serum Tg and TgAb levels on L-thyroxine and radiological examinations twice a year. The follow-up period was based on the date of death or the most recent contact as of May 31, 2024.

 DTC patients were examined clinically and treated according to the American Thyroid Association (ATA) clinical practice guidelines for thyroid cancer. Risk stratification of post-surgical DTC patients was performed according to the ATA guidelines. The diagnosis and histological classification of the tumors were performed in accordance with the World Health Organization (WHO) guidelines (1, 18). 

 Quantification of serum Tg, anti-Tg, and TSH levels was performed during monitoring intervals (every 6-12 months). Serum levels of TSH, Tg and anti-Tg were measured by electrochemiluminescence immunoassay method (Elecsys analyzer, Roche, Switzerland) at the Department of Biochemistry, Hospital 108.

### Diagnosis of lung metastasis

**Figure 2:**
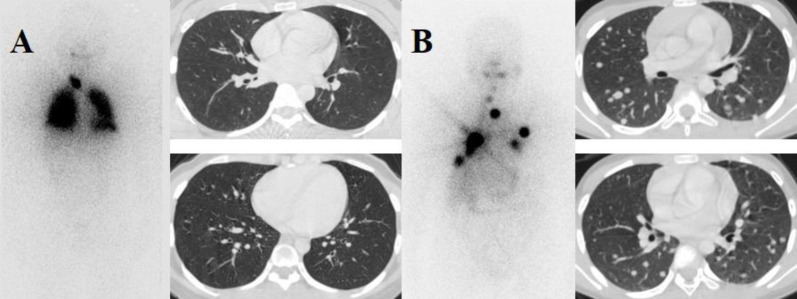
Representative patterns of lung radioiodine uptake in DTC patients with pulmonary metastases: (**A**) A 33-year-old female with lung metastases from papillary carcinoma of the thyroid. Diffuse pulmonary uptake on post-therapeutic ^131^I whole-body scan after the first treatment, without discrete pulmonary nodules on axial lung-window CT (miliary metastases). The patient underwent three therapeutic courses with a cumulative activity of 450 mCi, resulting in a decline in serum thyroglobulin (Tg) from 24.8 ng/mL to 0.29 ng/mL. (**B**) A 40-year-old female with lung metastases from papillary carcinoma of the thyroid. Focal pulmonary uptake on post-therapeutic ^131^I whole-body scan, corresponding to multiple scattered bilateral pulmonary nodules on axial lung-window CT. The patient underwent four therapeutic courses with a cumulative activity of 600 mCi, and serum thyroglobulin (Tg) decreased from 170.6 ng/mL to 0.89 ng/mL

Based on chest CT imaging and patterns of lung radioiodine accumulation observed on ^131^I WBS, DTC-LM patients were categorized into subgroups as follow: (i) DTC patients with negative chest radiography or CT results but positive lung ^131^I accumulation were defined as miliary pulmonary metastases, (ii) DTC patients with lung nodules ≤10 mm were defined as micronodular metastases and (iii) DTC patients with lung nodules >10 mm were defined as macronodular metastases. In addition, according to ^131^I whole-body scan findings, the patterns of lung radioiodine uptake were classified into three phenotypes: diffuse uptake, focal uptake, and negative uptake (Figure 2). DTC patients with lung metastasis only were defined as having no evidence of other metastases elsewhere (such as bone, brain, or liver). The presence of a thyroid remnant after surgery was not considered an exclusion criterion (6, 19, 20). The timing of lung metastasis diagnosis was used to classify patients into “initial” (diagnosed within 6 months of primary thyroid surgery) or “late” (diagnosed more than 6 months after surgery) (21, 22).

### Radioiodine WBS and Radioiodine therapy (RIT)

 Serum levels of thyrotropin (TSH), thyroglobulin (Tg) and antiTg were measured in all patients immediately prior to the administration of ^131^I, under a hypothyroid state induced by TSH stimulation (≥30 IU/mL). 

 This was achieved by withdrawing thyroxine (T4) at least 2 weeks and following an iodine restriction regimen (23). The patients underwent a pre-treatment radioiodine WBS after administering ^131^I, within 24 to 72 hours, with a dose ranging from 2-5 mCi (74–185 MBq). The post-treatment scan was performed 4 to 7 days after receiving the ^131^I treatment dose.

 Anterior and posterior planar parallel-hole collimator γ-camera images, from the top of the skull through the femurs, were acquired at a scan speed of 8-10 cm/min with a 256×1024 matrix, using the high energy of 364 keV and a 15-20% window. The WBS images are obtained through analysis and consensus of two experienced nuclear medicine physicians. Lung radioiodine uptakeis considered positive when the radioiodine accumulation is higher than the physiological uptake in the mediastinum (17). 

 DTC patients with lung radioiodine uptake were defined as the “positive” group. The patients without lung uptake were defined as the “negative” group. Thyroid tumour board including nuclear medicine physicians confirmed the sites of ^131^I accumulation using posttherapy WBS. Additional RIT was administered 6-9 months later to patients with ^131^I-avid metastases. All patients were treated with thyroxine to suppress TSH. Regular follow-up evaluations were performed every 6 months after surgery with clinical assessment and measurement of serum TSH, Tg, and Tg-Ab. Subsequent RIT was completed when ^131^I accumulation disappeared in lesions, new lesions appeared, or the attending physician decided to discontinue RIT due to the patient’s condition (21).

### Chest computed tomography (CT) imaging

 Chest CT, either without contrast (for evaluating lung parenchyma) or with contrast (for further assessment of the lymph node), were considered for DTC patients (DTC) at high risk, with elevated serum thyroglobulin (Tg) levels >10 ng/mL or elevated Tg antibodies, regardless of radioactive iodine (RAI) scan results are negative or not, or for patients with positive lung findings on imaging after initial treatment (1, 24). The chest CT was performed on multiple-slice CT scanners with a voltage of 120 kV, intensity of 140-200 mAs, spiral scanning with a slice thickness of 2.5 mm, pitch of 1.2, and images reconstructed in standard mode. CT images are processed and analyzed by a radiologist with over 5 years of experience. The diameter of pulmonary nodules was measured on axial CT images with a slice thickness of 2.5 mm. In accordance with RECIST 1.1 guidelines, the longest axial diameter of the two largest measurable nodules was recorded. Measurements were performed in the lung window setting, carefully aligned along the true longest axis on axial images, and were independently verified by an experienced radiologist. These measurements were assessed both before and after ^131^I therapy with a minimum follow-up interval of 6–12 months. Measurement and evaluation follow RECIST 1.1 guidelines (25).

***Evaluation of*** ^131^ ***I treatment response and clinical outcomes***

 Treatment response was classified into disease status as follows: the complete response

(CR) was defined as metastatic lesions have completely disappeared in post-treatment ^131^I WBS and/or CT, stimulated Tg levels <0.2 ng/ml in the thyrotropin (TSH) suppression state and below 1 ng/ml after stimulation, with thyroglobulin antibodies (TgAb) negative. Chest X-ray or CT does not detect any recurrence or metastasis after at least 12 months of follow-up; Partial Response (PR): post-treatment ^131^I WBS and chest CT showed the number of metastatic lesions has decreased with no new lesions, Tg levels were above 1.0 μg/L and have decreased by at least 25% compared to Tg levels before treatment. Stable disease (SD): post-treatment WBS, chest CT showed no new metastatic lesions, the size of existing lesions has not significantly changed, and serum Tg levels decreased by approximately 0–25% after treatment; Progressive Disease (PD): post-treatment WBS, chest CT showed an increase in the number and size of metastatic lesions,with new metastatic lesions that do not ^131^I uptake and serum Tg levels increasing by more than 25% after treatment (13). Based on disease status and mortality, DTC patients are categorized into non-progressive disease groups including complete response, stable disease, and partial response and progressive disease groups (progressive disease and death) (13, 26).

 Progression-free survival (PFS) is monitored and recorded from the time the patient is diagnosed with differentiated thyroid cancer with metastasis until the evidence of disease progression or death. Overall survival (OS) is monitored and recorded from the time the patient is diagnosed with DTC with metastasis until death from any cause. The follow-up period is up to the end of May 2024.

### Statistical analysis

 Data were analyzed using SPSS 22.0 software from IBM Corp and Medcalc 20.

 Quantitative variables were expressed as mean ± standard deviation (for normally distributed data) or median (for non-normally distributed data). Categorical values were compared using Chi-square or Fisher's exact tests. Continuous variables with normal distribution were compared using T-tests or ANOVA. Variables not following a normal distribution were compared using the Mann-Whitney U test. Kaplan-Meier curves were used to estimate PFS and OS based on the presence of clinical characteristics, CT imaging findings and ^131^I WBS. Differences between variables were assessed using the Log Rank test. Univariate and multivariate Cox regression analyses were performed to identify significant prognostic factors for disease progression and mortality. Statistical significance was defined as p<0.05.

## Results

The total number of patients with DTC-LM included in the study was 189. The mean age of DTC-LM patients in the study was 34.63±15.31 years (ranging from 7 to 73 years); 28 patients (14.8%) were aged ≥ 55 years; 69.8% were female. The majority of DTC-LM patients had papillary thyroid carcinoma, with an incidence rate of 89.4%.

 Of these patients, 67.7% had pulmonary metastasis diagnosed at the time of initial thyroid cancer diagnosis; 53.4% of DTC-LM patients did not show nodules on CT imaging (miliary metastases), while 78.3% had diffuse radiotracer uptake in the lungs.

 After total thyroidectomy, 95.2% of DTC-LM patients had elevated stimulated Tg levels, with a median Tg concentration of 336.26 ng/mL (range: 0.34–1000). The average number of ^131^I treatments was 6.37, with a mean cumulative dose of 873.47 mCi.

 The median follow-up time for overall survival (OS) was 110.57 months (range: 40-237). The progression and mortality rate among patients was 18.5%. The overall survival rates at 5 and 10 years were 96.83% and 94.71%, respectively.

 The progression rate was significantly higher in patients aged ≥55 years, those with follicular type, nodules sized 5–10 mm and >10 mm, and non-avid ^131^I nodules compared to the remaining DTC-LM patient groups (p<0.001) (Table 1).

**Table 1:** Clinical features in correlation with the progression of DTC-LM only patients

**Factor**		**Number of patients (%)**	**Non-progressive disease**	**Progressive disease**	**P**
**Gender**	Male	57 (30.2%)	46	11	0.856
	Female	132(69.8%)	108	24	
**Age(at diagnosis of LM)**	≥ 55	28 (14.8%)	12	16	<0.001
	< 55	161 (85.2%)	142	19	
	$\bar{X}$ ± SD (max – min)	34.63 ± 15.31 (73 – 7)			
**Pathology**	Papillary	169 (89.4%)	143	26	0.001
	Follicular	20 (10.6%)	11	9	
**Time to diagnosis of LM**	Initially	128 (67.7 %)	104	24	0.906
	Late	61 (32.3 %)	50	11	
**Pulmonary nodule size**	Miliary nodule	101 (53.4%)	98	3	<0.001
	< 5 mm	46 (24.3%)	38	8	
	5-10 mm	25 (13.2 %)	15	10	
	>10 mm	17 (9.1%)	3	14	
**Patterns of radioiodine uptake**	Diffuse	148 (78.3%)	131	17	<0.001
	Focal	24 (12.7%)	18	6	
	Non-iodine avid	17 (9.0%)	5	12	
**Stimulated serum Tg**	>10 mg/L	180 (95.2%)	147	33	0.769
	≤ 10 mg/L	9 (4.8%)	7	2	
	Median (min – max)	336.26 ± 302.27 (0.34 – 1000)			
**Number of ** ^131^ **I treatments**	< 5	48 (25.4 %)	36	12	0.181
	≥ 5	141 (74.6 %)	118	23	
	$\bar{X}$ ± SD (min – max)	6.37 ± 2.36 (3 – 14)			
**Cumulative ** ^131^ **I dose**	< 600 mCi	59 (31.2%)	45	14	0.214
	≥ 600 mCi	130 (68.8%)	109	21	
	Median (min – max)	873.47 ± 370.49 (120 – 2350)			
**PFS**	5 year	174 (92.06%)	154	20	NA
	10 year	160 (84.66%)	154	6	
	Median (min – max)	104.89 ± 48.69 (5 – 237)			
**OS**	5 year	183 (96.83%)	154	29	NA
	10 year	179 (94.71%)	154	25	
	Median (min – max)	110.57 ± 47.04 (40 – 237)			

**Figure 3:**
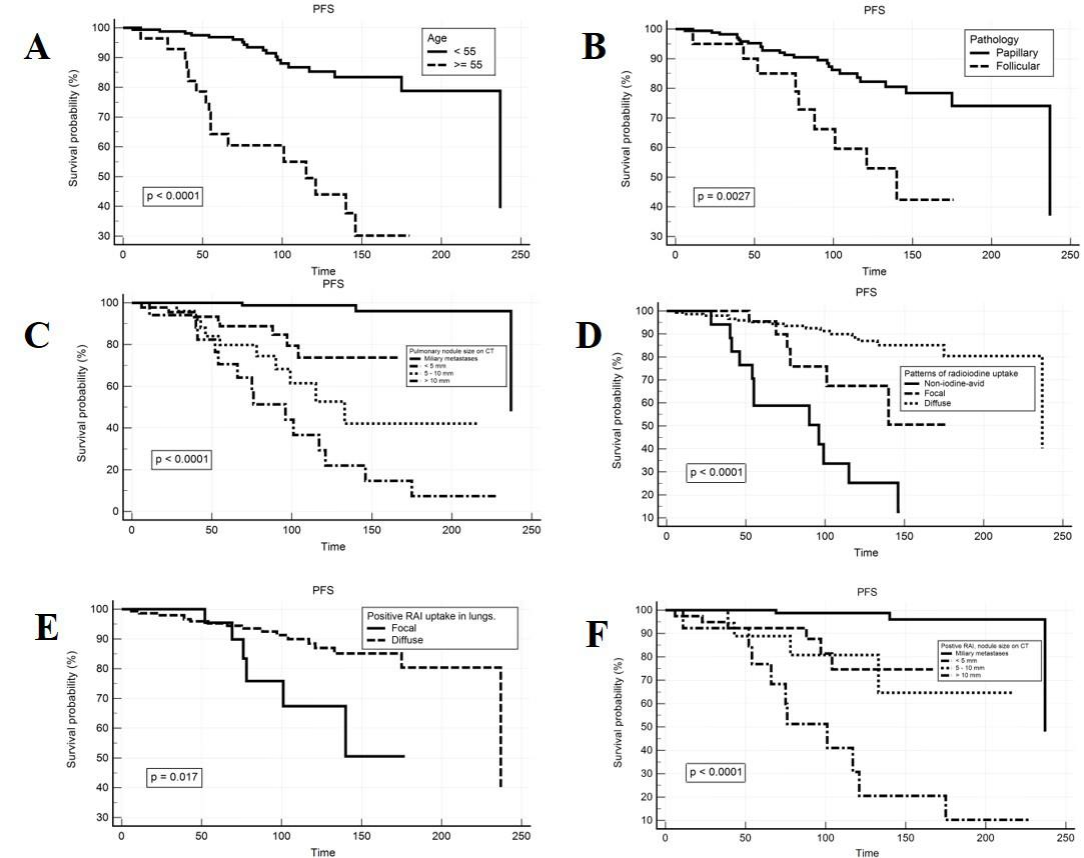
Kaplan-Meier curves showing the clinico-pathological, radiographic features in relation with progression-free survival (PFS). PFS is associated with age (**A**), pathological types **(B**), and the size of lung nodules on CT combined with ¹³¹I uptake pattern in the lungs on whole-body scan (**C**, **D**, **E**, **F**)

The 5-year and 10-year progression-free survival (PFS) rates for patients aged ≥55 are 64.3% and 49.5%, respectively, which are lower than the corresponding rates of 96.8% and 85.3% in patients aged <55 (Figure 3A). The cohort of differentiated thyroid cancer (DTC) with papillary histology exhibits 5-year and 10-year PFS rates of 92.8% and 82.2%, significantly higher than the corresponding rates of 85% and 59.6% observed in the DTC cohort with follicular histology (p=0.0027) as showed in Figure 3B.

 The group with diffuse lung ¹³¹I uptake demonstrates 5-year and 10-year PFS rates of 95.2% and 88.5%, compared to 89.8% and 67.4% in the group with focal ¹³¹I uptake (p=0.017) (Figure 3E). Conversely, the cohort with pulmonary metastasis non-^131^I avid presents higher progression rates, with 5-year and 10-year PFS rates of 58.8% and 25.2%, respectively (p<0.0001), as illustrated in Figure 3D.

Patients with DTC who do not have detectable nodules on CT (miliary metastases) show favorable treatment responses, with PFS rates of 100% and 98.8% after 5 years and 10 years, respectively, from the time of lung metastasis detection. This rate gradually declines to 88.8% and 73.8% in the group with nodules <5 mm; 79.8% and 52.7% in DTC-LM patients with nodules 5–10 mm; and the lowest PFS rates of 70.6% and 29.3% in the DTC-LM cohort with nodules >10 mm (p<0.0001) as showed in Figure 3C.

 The study results, as depicted in Figures 2C and 2F, indicate that nodules sized <5 mm and 5–10 mm (including both ¹³¹I uptake and non-uptake) exhibit 5-year PFS rates of 88.8% and 73.8%, respectively; and 10-year PFS rates of 79.8% and 52.7%, compared to the corresponding values for the uptake group of 92.3% and 74.6% versus 88.9% and 80.8% (p<0.0001).

 Univariate analysis in DTC-LM patients showed that diffuse ¹³¹I uptake in the lungs predicted a better treatment response with a higher PFS rate (p<0.001). In contrast, DTC patients with pulmonary metastatic nodule size >10 mm (whether with or without ¹³¹I uptake) predicted a higher risk of disease progression (p<0.001). Patients without detectable nodules on CT but with ¹³¹I uptake in the lungs were associated with a lower risk of disease progression, whereas the group with DTC-LM that did not exhibit lung ¹³¹I uptake combined nodule sizes ≥ 5 mm predicted a higher likelihood of disease progression (p<0.001).

 Multivariate analysis revealed that DTC-LM patients with diffuse ¹³¹I uptake or ¹³¹I uptake without detectable nodules on CT were predictive factors for a higher rate of progression- free survival (HR=0.378; CI=0.14–1.001; p=0.049 and HR=0.075; CI=0.017–0.34; p=0.005).

 In contrast, nodules larger than 10 mm, regardless of ¹³¹I uptake (HR=3.83; CI=1.59–9.25; p=0.003), and nodules ≥5 mm in the non-¹³¹I-avid group had a higher risk of disease progression (HR=6.58; CI=2.43–17.83; p<0.001) (Table 2).

**Table 2:** Univariate and multivariate Cox Regression Analysis of CT Imaging characteristics and ¹³¹I Uptake patterns in the lungs in relation to progression-free survival

**Imaging Characteristics**		**Univariate Analysis**			**Multivariate Analysis**		
		**HR**	**95%CI**	**p**	**HR**	**95%CI**	**p**
**Positive RAI and nodule size on CT**	Miliary metastases	0.038	0.009 – 0.158	<0.001	0.075	0.017 – 0.34	0.001
	< 5 mm	1.045	0.43 – 2.54	0.923	-	-	-
	5 – 10 mm	1.33	0.468 – 3.775	0.593	-	-	-
	> 10 mm	6.62	3.16 – 13.89	<0.001	3.96	1.69 – 9.24	0.001
**Negative RAI and nodule size on CT**	< 5 mm	2.23	0.534 – 9.35	0.271	-	-	-
	5 – 10 mm	9.27	3.74 – 22.97	<0.001	6.58	2.43 – 17.83	<0.001
	> 10 mm	8.38	2.93 – 23.92	<0.001	5.96	1.91 – 18.61	0.002
**¹³¹I Uptake Pattern**	Diffuse	0.18	0.09 – 0.36	<0.001	0.378	0,14 – 1.001	0.049
	Focal	1.814	0.75 – 4.39	0.187	-	-	-
	Non-iodine avid	7.85	3.84 – 16.03	<0.001	1.223	0.45 – 3.33	0.694
**Nodule size on CT**	Miliary metastases	0.038	0.009 – 0.16	<0.001	0.093	0.02 – 0.45	0.003
	< 5 mm	1.26	0.56 – 2.79	0.578	-	-	-
	5 – 10 mm	3.22	1.54 – 6.75	0.002	1.94	0.76 – 4.94	0.17
	> 10 mm	8.63	4.35 – 17.11	<0.001	3.83	1.59 – 9.25	0.003

**Figure 4:**
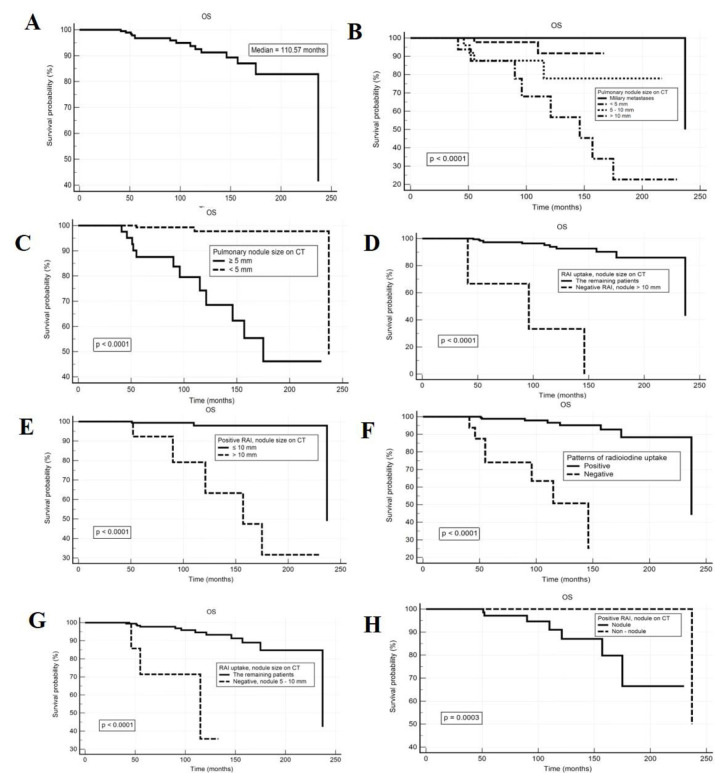
Kaplan-Meier curves showing the relationship between metastatic nodule sizes combined to ^131^I uptake paterns and overall survival. Overall survival rates of the entire DTC-LM patient cohort (**A**); OS and nodule size on CT (**B**, **C**); OS and pulmonary ^131^I uptake (**D**); OS, nodule size combined to ^131^I uptake in the lungs (**E**, **F**, **G**, **H**)

The median overall survival time for patients with DTC-LM is 110.57 months (Figure 4A).

 DTC-LM patients with ^131^I uptake in the lungs but without detected nodules on CT have a better prognosis compared to the group with positive scintigraphy and detected nodules on CT, with 5-year and 10-year OS rates of 100% and 100% versus 97.1% and 91%, respectively (Figure 4H).

 DTC-LM patients with positive scintigraphy and nodules larger than 10 mm have a poor prognosis, with 5-year OS at 92.3% and 10-year OS at 79.1%, significantly lower than the group with avid nodules but sizes <10 mm (99.4% and 97.9%), with p<0.0001 (Figure 4E). Nodules sized 5-10 mm that are non-avid for ^131^I and ^131^I-avid nodules >10 mm have markedly lower 5-year and 10-year survival times in the remaining patient group. Non-^131^I avid nodules >10 mm have the worst prognosis, with 5-year and 10-year OS rates of 66.7% and 33.3%, respectively (p<0.0001) as showed in Figures 4F, G.

 Disregarding ^131^I uptake on scintigraphy, nodules on CT sized ≥5 mm (5-10 mm and >10 mm) are associated with a poor prognosis, with 5-year and 10-year survival rates of 87.5% and 74.2%, significantly lower than the respective rates of 99.3% and 97.9% for the group with nodules <5 mm (p<0.00001) as described in Figure 4C.

 The results of univariate Cox analysis showed the best prognosis in the group of DTC-LM patients with positive RAI uptake in lung (positive RAI) but no nodules detected on CT, with a statistically significant increase in overall survival (OS) compared to other groups (p=0.04).

 Meanwhile, OS was significantly reduced in groups of patients with positive RAI and nodule size >10 mm (p=0.001), the group with negative RAI and nodules sized 5–10 mm (HR: 11.97; p<0.001), the negative RAI group (p<0.001), and the group with nodule size on CT >10 mm (p<0.001). The group with negative RAI and nodules sized >10 mm had the worst prognosis (HR: 23.63; p<0.001).

 In the multivariate analysis, independent prognostic factors associated with poor overall survival in DTC-LM patients included: (1) lung uptake of ^131^I with nodule size on CT>10 mm (HR: 5.91; p=0.018); (2) negative RAI with nodules sized 5–10 mm (HR: 12.85; p=0.002); nodule size >10 mm on CT (HR: 24.99; p<0.001); and (3) negative RAI uptake in lungs (nonuptaking nodules with any metastatic size detected on CT) with HR: 12.13; p=0.021. DTC-LM patients had a good prognosis with OS if nodule size was < 5 mm (HR: 0.078; p=0.003) (Table 4).

**Table 4:** Univariate and multivariate Cox analysis of scintigraphic characteristics and nodule size on CT with overall survival (OS)

**Imaging Features**		**Univariate Analysis**			**Multivariate Analysis**		
		**HR**	**95%CI**	**p**	**HR**	**95%CI**	**p**
**Positive RAI and nodule size on CT**	Miliary metastases	0.01	0.001 – 0.8	0.04	0.0001	0.001 – 0.27	0.922
	< 5 mm	0.38	0,05 – 2.93	0.352	-	-	-
	5 – 10 mm	0.7	0.09 – 5.33	0.727	-	-	-
	> 10 mm	6.98	2.32 – 21.03	0.001	5.91	1.36 – 25.69	0.018
**Negative RAI and nodule size on CT**	< 5 mm	3.28	0.423 – 25.52	0.255	-	-	-
	5 – 10 mm	11.97	3.15 – 45.47	0,000	12.85	2.53 – 65.41	0.002
	> 10 mm	23.63	6.35 – 87.94	<0.001	24.99	4.91 – 127.2	<0.001
**¹³¹I Uptake Pattern**	Diffuse	0.12	0.04 – 0.37	<0.001	0.59	0.07 – 4.99	0.63
	Focal	0.74	0.096 – 5.71	0.774	-	-	-
	Non-iodin-avid	19.13	6.04 – 60.59	<0.001	12.13	1.47 – 100.37	0.021
**Nodule size on CT**	< 5 mm	0.04	0.01 – 0.2	<0.001	0.08	0.014 – 0.42	0.003
	5 – 10 mm	2.84	0.89 – 9.07	0.078	-	-	-
	> 10 mm	14.21	4.91 – 41.14	<0.001	2.87	0.86 – 9.56	0.086

## Discussion

 Our retrospective study was conducted in largest cohort of DTC patients with pulmonary metastasis-only to determine the value of patterns of radioiodine uptake in correlation to metastatic pulmonary nodule size on CT in predicting progression- free survival (PFS) and overall survival (OS) for long-term management and follow-up in a major nuclear medicine center in Vietnam. In the present study, our results revealed that DTC patients with pulmonary metastasis-only have good long-term clinical outcomes. 5-year and 10-year overall survival rates were 96.83% and 94.71%, respectively. Clinical outcomes of DTC-LM patients were significantly correlated to patterns of radioiodine uptake and sizes of metastatic pulmonary nodules. Bilateral diffuse lung RAI uptake without pulmonary nodules detected on CT (miliary lung metastasis) was an independent predictor for favorable outcomes in DTC-LM patients. On the contrary, non-RAI uptake with any sizes of pulmonary nodules on CT (non-^131^I-avid pulmonary metastases), non-^131^I -avid pulmonary micronodules ≥5 mm and metastatic pulmonary macronodules (>10 mm) regardless of whether iodine-avid or non- iodine-avid are independent factors of predicting unfavorable OS in DTC-LM patients.

 The current study included 189 DTC-LM patients who received ^131^I therapy courses and periodically monitored with follow-up median of 110.57 months. PFS were significantly worse in DTC-LM patients aged ≥55, follicular subtype, non-^131^I-avid pulmonary micronodules 5-10 mm, macronodules (>10 mm) compared to remaining DTC-LM subgroups. 5-year and 10-year overall survival rates in DTC-PM patients in our present study were higher than those demonstrated in recent studies in Asian countries. SunWook Cho et al. analyzed in 152 DTC-LM patients in Korea during 11 years of median follow-up. They found that the 5- and 10-year survival rates of pulmonary metastatic DTC patients were 93% and 85% (5). Recently, Norihito Akatani et al. conducted retrospective study in series of 62 Japanese DTC-LM patients with a median follow-up of 7.9 years (21). Their results have demonstrated that the 5- and 10-year OS rates were 93% and 72%, respectively. 

 In contrast to these previous studies in which metastatic pulmonary and extrapulmonary DTC patients were enrolled, our present study included patients with lung metastasis alone (5, 6). Several previous studies showed that the benefits of RAI therapy on postoperative DTC patients with lung metastasis alone better to those DTC-LM combined with other extra-pulmonary distant sites (6, 27). Furthermore, these heterogeneity in demographic characteristics and clinico-histopathological features (male/female ratio, age at DTC-LM diagnosis, size of the primary cancer, follicular or papillary subtype, timing of metastasis, multiple sites of metastasis in complex study cohorts may be attributed to differences in survival rates from study to others (28-31). In our current study, we focused on analyzing patterns of lung radioiodine uptake in relation to pulmonary nodule size for predicting long-term survival rates of DTC patients with lung metastasis only.

 Although conventional gamma camera is not ideal for imaging high energy radionuc lide as ^131^I, radioiodine planar WBS is mainstay functioning imaging in diagnosis, predicting ^131^I treatment response and prognosis as well in clinical practice (19, 32, 33). Our data showed that radiographic patterns of lung radioiodine uptake significantly correlated to PFS. 

 Univariable analysis of DTC-PM subgroups revealed that pattern of bilateral lung diffuse radioiodine uptake was radiographic finding correlated significantly to ^131^I therapy efficacy. Consequently, these lung uptake patterns were an independent factor for predicting a favorable PFS (HR=0.378; CI: 0.14–1.001, p=0.049) in multivariable analyses. We also found that the patterns of diffuse lung radioiodine uptake correlated to OS in the univariate analysis. However, the lung ^131^I -avid uptake finding was not independent predictor for favorable OS in the multiple Cox analysis (p=0.618).

 Conversely, non-^131^I -avid pulmonary metastasis predicted low OS in DTC-LM patients. In our series of DTC-LM patients, we found that non-^131^I -avid metastatic lesions related directly to either fail to uptake iodine or lose iodine uptake properties following ^131^I treatment courses eventually develop radioiodine-refractory thyroid cancer. Additionally, a number of previous studies reported that there was an association with age, biological behavior of pathological subtypes, metastatic site which correlated to tumor differentiation and radioiodine-avid ability (30, 31). In the present study, the results demonstrated that PFS were significantly worse in DTC-LM patients with aged ≥55, follicular subtype. The loss of iodine uptake may be consequent upon the dedifferentiation of pulmonary metastases resulting in reduced expression of the sodium/iodide symporter (NIS) that associated with a poor clinical outcome (13, 31, 33). Hong-Jun Song et al. found that DTCLM patients unresponsive to RAI therapy has a significantly lower 10-year survival rate (6).

 As reported previous in the literature, lung radioiodine uptake on ^131^I-WBS but negative chest CT findings were radiographic features commonly presented in DTC-LM patients. In general, the size of metastatic pulmonary nodules under 2 mm, defined as pulmonary military metastasis were practically not visualized on conventional CT imaging. The military pulmonary radioiodine-avid metastases were invariably related to good ^131^I uptake. A number of previous studies revealed a significant correlation between ^131^I treatment response in these DTC-LM subgroups (6, 13, 29). In our study, the analysis indicated that lung ^131^I uptake without pulmonary nodules detected on CT (miliary metastasis) was an independent indicator for favorable PFS in DTC-LM patients. 5-year and 10-year PFS were 100%, 98.8% and 10 year survival rate of up 100% in DTC patients with miliary pulmonary metastases.

 Of those DTC-LM patients, pulmonary nodules detected on CT were categorized into metastatic macronodular and micronodular subgroups. When analysing correlation between metastatic pulmonary nodule sizes and clinical outcomes, our analysis indicated that ^131^I-avid nodular metastases reaching threshold over 10 mm correlated significantly to disease progression in DTC-LM with lung ^131^I uptakes. Macronodular pulmonary metastasis was significantly correlated with a reduced survival rate. In those with non ^131^I avid pulmonary macrometastases, 5-year 10-year survival rates were 66.7% and 33%, respectively (p<0.001). The DTC patients with metastatic pulmonary sizes >10 mm regardless of whether ^131^I-avid or non-^131^I-avid were more likely to increased progression rate compared to remaining subgroups (HR=3.83; CI =1.59–9.25; p=0.003). Non ^131^I -avid subcentimeter micrometastases even from ≥5 mm in size increased likelihood of disease progression and deaths (HR=13.23; p=0.002). 

 Interestingly, our results showed that non-^131^I -avid pulmonary nodular micrometastasis ≥5 mm in size was an independent factor associated with poor progression- free survival (HR=6.162; CI: 2.314–16.413; p<0.001), whereas in the ^131^I -avid group, only nodules >10 mm were found to be independent prognostic factors (HR: 3.96; CI: 1.69–9.24; p=0.001).

 There are some limitations in our present study. Firstly, the study had a retrospective design. During the long-term follow-up period, some clinical data involved to TNM stage, variant pathological subtypes were missing and several clinico-histopathological features in latest ATA risk stratifications, gene mutations not included could be affected to clinical outcomes and may have caused a bias in the data selection. Secondly, during the diagnosis of non-^131^I-avid metastatic lesions, histopatho-logical confirmation was not always feasible. Finally, micronodular pulmonary metastases under resolution threshold of conventional gamma camera, particularly for patients with partial ^131^I-avid metastatic lesions and partial non-^131^I-avid could be not detected on planar WBS imaging. In this clinical scenario, adoption of SPECT/CT in such patients with pulmonary micrometastases could provide equivalent or even incremental information from further management viewpoint. Hence, a further prospective study addressing these clinical issues is needed, which would have more practical implications.

## Conclusion

 In summary, our study indicated that DTC patients with pulmonary metastasis-only have good long-term clinical outcomes that were significantly correlated to patterns of radioiodine concentration and sizes of metastatic pulmonary nodules. Lung metastases with bilateral diffuse radioiodine accumulation correlated significantly to ^131^I therapy response rate and was an independent factor for predicting a favorable PFS in DTC-LM patients. Miliary lung metastasis from DTC-LM only was independent predictor for favorable PFS and correlated to OS. On the contrary, predictive factors including non-^131^I-avid pulmonary metastases with any size, non-^131^I-avid pulmonary micronodules ≥5 mm and metastatic pulmonary macronodules are independent indicators in predicting unfavorable OS in these patients with DTC-LM alone. Further well-designed prospective studies using radioiodine SPECT/CT may help for providing more clinical implications in those DTC-LM patients.

## Abbreviations

**DTC:** Differentiated Thyroid Carcinoma

**DTC-LM:** Differentiated Thyroid Carcinoma with Lung Metastasis

**RIT:** Radio Iodine Therapy

RAI: Radio Active Iodine

WBS: Whole-Body Scan

CT: Computed Tomography

TSH: Thyroid-Stimulating Hormone

Tg: Thyroglobulin

Tg Ab: Thyroglobulin Antibody

HR: Hazard Ratio

CI: Confidence Interval

CR: Complete Response

PR: Partial Response

SD: Stable Disease

PD: Progressive Disease
